# Dr. Isaac J. Wetherbee

**Published:** 1902-09

**Authors:** 


### Obituary.

### DR. ISAAC J. WETHERBEE.

THE following members of the Board of Trustees of the Boston Dental College have learned with regret of the death of Dr. Isaac J. Wetherbee, a member of this board since it was organized as a corporation in 1868, and its first president, which office he held almost continuously from the time of such organization until 1899, when, in consequence of the changed conditions and methods of dental education, it seemed wise to suffer the college to become the Dental Department of Tuft's College. In recognition and remembrance of the unremitting constancy and devotion with which Dr. Wetherbee during this long period of time performed his official duties, this board has caused this minute to be made upon its records.

W. P. LEAVITT,
S. G. STEVENS,
B. S. LADD,
*Committee.*

BOSTON, June 26, 1902.

